# Women’s experiences regarding the care received in the hospital setting during the abortion process

**DOI:** 10.1590/0034-7167-2025-0521

**Published:** 2026-10-16

**Authors:** Mariana Enumo Balestre, Iven Giovanna Trindade Lino, Maria Eduarda Pascoaloto da Silva, Juliana Amaral Prata, Telmara Menezes Couto, Viviane Cazetta de Lima Vieira, Erika dos Santos Ratuchnei Dal Pizzol, Sonia Silva Marcon

**Affiliations:** I Universidade Estadual de Maringá. Maringá, Paraná, Brazil; II Universidade Estadual do Rio de Janeiro. Rio de Janeiro, Rio de Janeiro, Brazil; III Universidade Federal da Bahia. Salvador, Bahia, Brazil

**Keywords:** Abortion, Spontaneous, Attitude of Health Personnel, Professional-Patient Relations, Empathy, Qualitative Research., Aborto, Mujeres, Atención Hospitalaria, Humanización de la Atención, Enfermería.

## Abstract

**Objectives:**

to understand how women recall the care they received in the hospital setting during the abortion process.

**Methods:**

a descriptive qualitative study grounded in the conceptual framework of the National Humanization Policy. Data were collected between May and September 2024 through audio recorded interviews, conducted either in person or remotely, with 19 women, and analyzed using thematic content analysis.

**Results:**

two categories emerged: Experiencing disrespectful care, which reflects dissatisfaction with communication, insufficient information, devaluation of complaints and emotional suffering, lack of privacy, and hospitalization alongside pregnant women; and Positive care experiences, which include humanized care, emotional support, and active listening.

**Final Considerations:**

women generally recall the care received in a negative way, highlighting the need for adopting and implementing good obstetric practices and reinforcing the guidelines of the National Humanization Policy in care provided during the abortion process.

## RESUMEN

**Objetivos:**

aprehender cómo las mujeres rememoran la atención recibida en el contexto hospitalario durante el proceso de abortamiento.

**Métodos:**

estudio descriptivo cualitativo, basado en el marco conceptual de la Política Nacional de Humanización. Los datos fueron recolectados entre mayo y septiembre de 2024 mediante entrevistas de audio grabadas, presenciales o remotas, con 19 mujeres, sometidos a análisis de contenido.

**Resultados:**

surgieron dos categorías: Viviendo una atención irrespetuosa, que evidencia insatisfacción con la forma de comunicación, información insuficiente, desvalorización de las quejas y del sufrimiento emocional, falta de privacidad y hospitalización conjunta con gestantes; y Experiencias positivas en el cuidado, que incluyen atención humanizada, apoyo emocional y escucha activa.

**Consideraciones Finales:**

las mujeres rememoran la atención recibida de manera negativa, lo que señala la necesidad de adoptar e implementar buenas prácticas obstétricas y de valorar las directrices de la Política Nacional de Humanización en la atención durante el proceso de abortamiento.

## INTRODUCTION

Abortion is a relatively common event in women’s reproductive trajectories^(1)^. In Brazil, it constitutes a public health issue, given the total of 2,258,104 hospitalizations due to abortion between 2008 and 2018, representing an annual average of 37.4 hospitalizations for every 10,000 women of reproductive age^(2,3)^. Moreover, of the 19,535 maternal deaths recorded in the country between 2012 and 2022, 663 (3.39%) were related to abortion^(4)^. These data highlight the magnitude of the problem and underscore the need for a critical analysis of how care for women experiencing abortion has been conducted.

In such cases, hospital care can influence the recovery process, especially when attitudes and practices reveal negligence, discrimination, and dehumanized behaviors, which unfortunately are common in obstetric settings^(2,5)^. Care for women during this process must encompass clinical, psychological, social, and ethical dimensions^(6)^, as there is evidence of significant impacts on physical and mental health^(7)^. The experience of abortion may be marked by intense grief and frequent severe grief reactions - generally decreasing over time^(8)^ - as well as increased risk of anxiety, depression, and post traumatic stress disorder, which are more common in cases of recurrent spontaneous abortions^(9)^.

Given the repercussions of the abortion process on women’s physical and mental health, the relevance of ensuring that hospital care encompasses clinical, psychological, social, and ethical dimensions becomes evident. A systematic review including studies conducted in Asia, Africa, Australia, and Europe found that many women with a history of spontaneous abortion develop mental disorders such as anxiety, depression, and stress within the six weeks following the event, and that this is more frequent in low and middle income countries^(10)^. These effects were also observed in a study conducted in Brazil, which identified higher prevalence among first pregnancy and younger women^(11)^.

In light of these issues, it is essential to guarantee women’s right to dignified care and to ethical, empathetic, and effective support. To this end, the normative and programmatic discourse of the Brazilian Ministry of Health reinforces the importance of considering the person in their entirety, recognizing emotional, social, and cultural needs as essential components of comprehensive health care. Accordingly, the National Humanization Policy (PNH), established in 2013, sets forth guidelines that emphasize welcoming practices, qualified listening, the strengthening of bonds between professionals and users, and shared responsibility in care^(12)^.

Despite this cross cutting public policy within the *Sistema Único de Saúde* (SUS, Brazilian Unified Health System), providing humanized care in situations of abortion remains a challenge, whether due to professional prejudice, work overload, lack of adequate training, or weaknesses in service infrastructure^(13)^. As a result, care is often limited to a clinical and procedural approach, characteristic of the biomedical model, disregarding the subjectivities and social aspects that shape the abortion experience, with the potential to culminate in obstetric violence^(13,14)^.

In Brazil, scientific production in this field is predominantly focused on epidemiological and normative analyses, as well as on the physical and mental repercussions of the event^(2,7)^. Although essential, these studies offer only a limited exploration of how hospital care during the abortion process is perceived by women, which restricts understanding of the gaps between the care recommended in public policies and the care actually provided. By giving visibility to the experiences lived by these women, the present study contributes to deepening the subjective and relational dimensions of care, supporting qualified care practices, continuing education initiatives, and the implementation of good obstetric practices.

This article is the product of a master’s thesis in Nursing defended in March 2025^(15)^.

## OBJECTIVES

To understand how women recall the care they received in the hospital setting during the abortion.

## METHODS

### Ethical aspects

The study complied with the requirements of Resolutions No. 466/2012 and No. 510/2016 of the *Conselho Nacional de Saúde* (Brazilian National Health Council), as well as the guidelines for research conducted in virtual environments (National Research Ethics Commission/2021) and was approved by the Research Ethics Committee for Human Subjects of the sponsoring institution (Opinion No. 6.656.413).

All participants provided consent to take part in the study by signing the Informed Consent Form (ICF). In the case of remote interviews, the ICF was previously sent via WhatsApp and returned as a scanned, duly signed digital file. To ensure participants’ anonymity, in the presentation of the study results, excerpts from their statements are identified with the letter E, referring to “interviewee”, followed by a number indicating the order of inclusion in the study, as well as additional indicators corresponding to the number of pregnancies (G), abortions (A), and live births (NV).

### Type of study and theoretical framework

This is a descriptive, exploratory qualitative study that used the National Humanization Policy (NHP) as its conceptual framework. This approach allows for the exploration of participants’ lived experiences and subjective perceptions, especially regarding sensitive topics^(16)^, such as the abortion process.

The PNH, also known as “*Humaniza SUS*”, aims to support the implementation of the principles of Brazil’s Unified Health System (SUS) in the daily routine of health services, promoting changes in management and care practices. In the context of care for women during pregnancy, childbirth, and the postpartum period, several key guidelines stand out, namely: welcoming practices, the care environment, expanded and shared clinical practice, and the defense of users’ rights^(12)^. It is important to note that the preparation of the research report followed the recommendations of the Consolidated Criteria for Reporting Qualitative Research (COREQ)^(17)^.

### Methodological procedures

The study participants were selected by convenience, and recruitment took place through a review of medical records, followed by in person contact in the gynecology clinic of a public hospital with 30 obstetric beds and an emergency service for obstetric complaints. Given the low initial participation (five women), additional strategies were adopted, such as disseminating the study in a support group for bereaved parents, which holds biweekly online meetings coordinated by a psychologist (two women), and posting a one minute video on Instagram^®^ in which the principal researcher introduced herself and explained the motivation, criteria, and procedures for participating in the study (12 women). In all cases, the researcher shared her phone number and email address for further contact.

The predefined inclusion criteria were being 18 years of age or older, having received hospital care during the abortion process - regardless of type (induced or spontaneous), procedures performed, or time elapsed since the event. Women who reported not feeling emotionally prepared to discuss the topic were not included.

During the initial contact, all potential participants were informed about the study’s objectives, motivation, and the type of participation expected. Those who agreed to participate were asked to provide a phone number to schedule the interview. Of the 26 women contacted, five were not included because they did not feel comfortable recalling the experience, despite the long time elapsed since the event (as identified in their medical records), and two did not respond to subsequent contact attempts, resulting in a total of 19 participants.

### Data Collection

Data were collected between May and September 2024 through individual semi structured interviews, scheduled on the day, time, and location preferred by the participant and according to the researcher’s availability. All interviews were conducted by one of the authors (a nurse, single, master’s student in nursing, with no experience in caring for women undergoing abortion but with expertise in qualitative data collection), who had no prior relationship or contact with the participants.

It is important to clarify that 11 interviews were conducted in person (nine in the participants’ homes and two in a private room at the hospital), and eight were conducted remotely via Google Meet^®^. With participants’ consent, all interviews were audio recorded using a mobile phone (for in person interviews) or the videoconferencing platform (for remote interviews). They lasted between 30 and 105 minutes (average of 55 minutes), without interruptions or incidents that compromised the quality of the recordings or the narratives. Each participant was interviewed only once, and all interviews took place in the presence of only the participant and the researcher, except for one case in which the participant’s partner was at home but remained in another room for most of the interview.

To conduct the interviews, an interview guide was developed and tested with four women, whose interviews were not included in the study due to adjustments made to the instrument. These adjustments involved: (i) adding questions about sociodemographic characteristics and support questions to strengthen the study’s lines of inquiry, and (ii) reformulating some questions that had evaluative wording. After these modifications, the guide consisted of two parts: the first with questions about sociodemographic characteristics (age, race, marital status, number of living children, number of abortions), and the second composed of a guiding question: “Tell me about how you experienced the abortion process and how the care you received was during that time”. Some supporting questions were also used when necessary, such as: “What would you have liked to be different during this process? Why? Specifically regarding the care you received, what do you identify as positive? And negative? How was your interaction with the medical and nursing teams? And with other patients? Did you participate in decisions related to your treatment? In what way? Do you feel you had the freedom to ask questions and request clarification? Is there anything else you would like to say about your experience?”.

At the end of each interview, in a private setting, the researcher recorded on her mobile phone her impressions regarding the context and nonverbal behaviors (facial and bodily expressions, voice tone, pauses, silences, crying). Preferably on the same day the interview was conducted, the content and the researcher’s observations were fully transcribed with the support of Transkriptor^®^. During this process, no doubts emerged that would justify recontacting the participant. The transcribed material was sent only to the participant who expressed interest in reading the transcript for possible corrections, deletions, or additions. However, she did not provide any comments or indicate the need for corrections. All other participants explicitly stated that it was unnecessary to review the transcript, given that the interview had been audio recorded.

For concluding data collection, the criterion of data sufficiency and exhaustion was adopted^(18)^. Theoretical saturation was identified in the sixteenth interview, at which point no new codes or analytically relevant elements related to the phenomenon under investigation emerged. To confirm the repetition of information and ensure the consistency of the findings, three additional interviews were conducted, totaling 19 participants, without compromising theoretical saturation.

### Data Analysis

The information regarding sociodemographic and obstetric characteristics was organized in an Excel spreadsheet and analyzed descriptively using absolute frequencies. For the qualitative data, no software was used; instead, the transcribed interview content was subjected to thematic content analysis, following Bardin’s approach^(19)^, developed in three stages.

In the pre analysis stage, the empirical material was organized and the researcher became familiar with it through a floating reading of the interviews, with the aim of grasping the overall set of narratives and defining the analytical corpus. At this stage, the criteria of exhaustiveness, representativeness, homogeneity, and relevance of the data were considered.

In the material exploration stage, the units of meaning present in the participants’ statements were identified, expressing recurrent aspects of their experiences related to the care received during the abortion process. These excerpts were manually coded and subsequently grouped into initial themes according to the convergence of meanings observed in the narratives. From this analytical process, the themes were organized into analytical categories, as presented in Chart 1.

**Chart 1:** Definition of recording units, thematic axes, and analytical categories of the study

Record entry (n)	Themes	Categories
- First impressions upon arrival at the service (n=13) - Characteristics of the initial reception (n=14)	Initial reception and approach	Experiencing disrespectful and limited care during the abortion process
- Normalization stance regarding the absence of fetal heartbeats (FHB) (n=3) - Manifestations of professionals blaming the pregnant woman (n=1) - Complaints about medical care (n=7)	Impersonal professional conduct and violence	
- Perceptions about sharing physical space with women in labor or postpartum women with their babies (n=7) - Descriptions of explicit traumatic episodes (n=17)	Exclusion and suffering	
- Positive remarks about the care received (n=6) - Mentions of being placed in a separate ward (n=1) - Experiences related to multiprofessional care (n=12)	Welcoming experiences and sensitive care	Welcoming and humanized experiences in moments of vulnerability

Finally, in the stage of results processing, inference, and interpretation, a critical analysis of the categories was carried out, seeking to understand the meanings attributed by the women to their experiences. This analytical process was guided by the principles and guidelines of the National Humanization Policy^(12)^, articulating the empirical findings with the scientific literature and the institutional context of hospital care, which made it possible to identify weaknesses and strengths in the care provided, as well as to point out pathways for improving assistance during the abortion process.

To ensure credibility, dependability, confirmability, and transferability, the entire research process - with emphasis on methodological description - was documented and is available for future consultation. Additionally, during the analysis, efforts were made to identify internal coherence within the narratives, thematic recurrence, and interpretative consistency.

## RESULTS

The 19 women who participated in the study were between 21 and 56 years old; among them, 17 reported having a partner and 14 reported having living children. Sixteen identified themselves as white, and more than half (10) reported receiving care through private health insurance during the abortion process. Eleven participants lived in the study municipality, five in other municipalities in the state of Paraná, two in other states, and one in Osaka, Japan.

A total of 31 abortions were reported (28 spontaneous, one induced, and two legal), with the number of abortions per woman ranging from one to four. The time elapsed between the last abortion and the interview ranged from six months to 28 years. From the analysis of the interviews, two categories emerged, which are described below.

### Experiencing disrespectful and limited care during the abortion process

Some women reported that their dissatisfaction with the care received began as soon as they arrived at the health service, in the reception area, where risk classification is performed:

*I arrived, and the triage nurse asked how heavy my bleeding was, and I said two. She said: “Oh, that’s still little! So I’m going to classify you as green”. And I stayed there the whole morning waiting to be seen*. (E10 - G1/A1/NV0)

*I had active bleeding! I made that very clear. Maybe I imagined things, but it seemed like my skin color influenced the way they treated me. They classified me as green, with active bleeding and 5 weeks pregnant!* (E12 - G1/A1/NV0)

From the clinical investigation to the confirmation of the abortion, the participants’ accounts reveal concerns regarding communication with the healthcare team, which was marked by impersonal medical interactions, a lack of explanations, or the provision of incomplete information.

*I didn’t feel that I received enough information or that they made me feel safe. “Look, this is what happened. We’re going to do the curettage, there’s no way not to do it”, or something like that. They didn’t give me any choice at any moment*. (E15 - G2/A2/NV0)

*I was in Japan, and they just told me I was miscarrying. I stayed at the clinic until they discharged me.* […] *They didn’t tell me what could have happened, they didn’t explain anything, they just left me there until they thought my tests were good enough for me to be discharged.* (E16 - G2/A1/NV1)

*It was my first pregnancy. I was with my husband, and the doctor was very cold in the way he spoke. During the transvaginal ultrasound, he said: “Look, your pregnancy didn’t progress”. And nothing else. I couldn’t even ask anything* […]. *The only thing they gave me was a paper about the risks of the surgery. I didn’t even know it would be general anesthesia. I thought I would stay awake, but I blacked out. I have no idea what happened in that room.* (E17 - G1/A1/NV0)

The lack of privacy during care - marked by the presence of several students, repeated examinations, and the absence of prior explanations before procedures - made the women feel uncomfortable, violated, exposed, and disrespected.

*The doctor came in and said: “Open your legs!”. I was like, huh? Because, either way, I felt violated! Even my husband, who was sitting in the chair, was startled.* (E6 - G4/A2/NV2)

*I stayed, I think, about 40 minutes in the gynecological position. One would come and look* […]. *Especially because it’s a university hospital! Then another came, and another* […]. *It was uncomfortable!* (E5 - G5/A3/NV2)

*I witnessed some things that were unpleasant. Not only because I’m a woman and pregnant. I think that just by being in a hospital, I should have been treated with respect, and with privacy, which I didn’t have. I was seen with several people in the room.* (E14 - G2/A1/NV1)

The fact that they remained hospitalized while awaiting procedures such as curettage or manual vacuum aspiration, in the same ward as women in labor or postpartum women with live newborns, was a source of suffering for the participants

That night was distressing for me because in that room there were only pregnant women. There were women listening to their baby’s heartbeat, another one with that little device because she was about to have a C section […]. *That destroyed me! I just cried because I was going in for surgery to remove mine, and they were going to have their babies.* (E17 - G1/A1/NV0)

*I looked to one side, there was a pregnant woman; I looked to the other, there was a woman with a huge belly, or with a baby, and I was alone* […]. (E12 - G1/A1/NV0)

[…] *I was in the maternity ward, and all the time someone would walk by asking when I was going to give birth. I already knew there was nothing inside me anymore.* (E18 - G5/A4/NV1)

In situations of illegal abortion, embarrassment and fear were very present from the moment the women arrived at the hospital.

It was a very difficult decision. It’s not something accepted, it’s illegal and all that. I didn’t know if I would have any problems, but for me it really didn’t make sense at that moment. So, besides the choice itself, there was the worry of being in front of a doctor, having to lie, dealing with that whole situation […]. *Whether we like it or not, we never know who we’re going to come across* […]. (E11 - G1/A1/NV0)

In addition to the negative feelings experienced, some interviewees reported negligence in care that resulted in unfavorable outcomes, causing further suffering

*They didn’t perform the curettage correctly because they said my uterus was very swollen. I was hospitalized because they wanted to do another curettage. However, after that care, they didn’t do another ultrasound to see how things were. To check if it had really been cleaned, if the whole placenta had come out* […]. *I ended up losing my uterus because the placenta became infected inside me. I had hemorrhage, sepsis, and I ended up losing my uterus.* (E2 - G1/A1/NV0)

*I had a severe infection and retained placental tissue. I hadn’t expelled everything because my cervix had closed again. I was discharged, but I had to return to the hospital to undergo more tests and another curettage. That whole agonizing process all over again!* (E1 - G2/A1/NV1)

*Before the third miscarriage, no ultrasound was done because the doctor who performed them wasn’t at the hospital, and he thought it wasn’t necessary. I lost that one, again* […]. (E4 - G4/A4/NV0)

### Welcoming and humanized experiences in moments of vulnerability

Some women recalled having received humanized care, expressed through welcoming interactions, sensitive attention, and the guidance they were given about the abortion process:

*She did the pelvic exam and said: “Your cervix is open!”. I thought, “This isn’t menstruation!” and I asked, “Is something happening, doctor? Is something wrong?”. She told me to get dressed first. Today, I see that as a sign of care, right?* (E18 - G5/A4/NV1)

*Once again, I was at the hospital. I was welcomed in a way I had never been before. It was my 4th pregnancy, the staff already knew me, all the nurses talked to me* […]. *Everything was very comforting! I stayed there with them for a little while, and it was very gratifying for me. Besides that, I received a lot of support from the doctor who did the ultrasound. He was very thorough in his explanations.* (E4 - G4/A4/NV0)

Positive perceptions were associated with professionals’ expressions of availability and empathy, active listening, and respectful attitudes, as well as with the provision of information, clarification of doubts, and psychological support during hospitalization

*They came to check on me all the time to see if I was okay. There was a doctor who gave me a lot of support when I needed it. The nurse too. They talked to me a lot. The doctor even said she would check my tests faster so I wouldn’t have to stay there too long. She would speed up my discharge* […]. *I felt they gave me all the support I needed.* (E8 - G1/A1/NV0)

*The nurses were very sensitive. When the anesthesiologist arrived, I said: I just don’t want my arms to be tied down, I want to put my hands on my head. That position reminded me of the previous curettage. And he allowed it, he was very understanding, I will never forget that.* (E9 - G2/A2/NV0)

The psychologist would come talk to me every day! [...] she came with words, but also to listen to me. She stayed a long time with my husband, taking care of him. (E2- G1/A1/NV0)

The place of hospitalization - whether in units outside the maternity ward or in rooms separated from other pregnant or postpartum women - was a key factor in making the experience less traumatic.

*I had to wait until the next day to have the ultrasound. They didn’t keep me in the maternity ward because they thought I was going to lose it.* (E2 - G1/A1/NV0)

*I stayed in the maternity ward, but next to me was a woman who had had her uterus removed. Thank God she wasn’t pregnant or postpartum.* (E4 - G4/A4/NV0)

*I told the doctor, “I don’t want to stay in the maternity ward”, and she said, “There’s no way, because to induce your labor, you have to stay”. There were all those pregnant women, and I started saying, “Get me out of here!”. Since I had private insurance, I was able to go to another hospital, where I wasn’t placed in the maternity ward. It was the best decision I could have made at that moment.* (E1 - G2/A1/NV1)

## DISCUSSION

In general, the women in this study recalled that the hospital care they received during the abortion process was characterized by delays in the initial assessment, impersonal professional interactions, communication problems, exposure of their bodies, lack of explanations and of prior consent for examinations and procedures, negligence, and suffering resulting from being placed in shared wards with pregnant or postpartum women. In other words, for these women, the abortion process was marked not only by pregnancy loss but also by negative care experiences that intensified their pain and suffering.

These findings run counter to the guidelines of welcoming practices, qualified listening, assurance of privacy, and shared responsibility in care as recommended by the National Humanization Policy^(12)^. At the same time, they reveal situations that violate women’s rights to dignity, information, privacy, and autonomy, and highlight the invisibility of the subjective dimensions that permeate this reproductive experience - particularly considering that most participants had experienced more than one spontaneous abortion.

In such situations, it is known that emotional support and the relationships established with health professionals can influence the abortion experience either positively or negatively^(20)^. In line with the findings of this study, a systematic review found that women’s dissatisfaction with care provided in emergency services in Pennsylvania and other regions of the United States was due, in part, to the lack of emotional support^(21)^.

Given that the support received during the abortion process shapes the lived experience and the associated psychological impact^(9)^, it is important for professionals to consider women’s lack of knowledge about the parameters used for risk classification (amount of blood loss, heart rate, blood pressure, gestational age, and pain intensity). They should inform women that although pregnant individuals with bleeding are prioritized for care^(22)^, the waiting time will not worsen their clinical condition. However, considering that welcoming practices involve recognizing users’ unique needs and life contexts, neglecting this guideline reinforces the mechanical application of technical norms and the development of reductionist practices that intensify the psychological repercussions of pregnancy loss^(12)^.

In this regard, a systematic review identified that women in Asia, Europe, and Africa reported high levels of anxiety, depression, and stress related to the experience of spontaneous abortion^(10)^. Among Norwegian women, greater psychological distress was observed after spontaneous abortion in the short and medium term, whereas induced abortion was associated, in the long term, with higher scores of avoidance, guilt, shame, and relief^(23)^.

For these reasons, clear communication between professionals and patients is essential, as it facilitates the collection of relevant information for developing the therapeutic plan, promotes well being, and strengthens the ethical principles of non maleficence and autonomy - provided it is grounded in active listening, non stigmatizing language, and recognition of women’s suffering and rights^(24-27)^. These characteristics were not identified by the participants in their interactions with professionals, as they often resorted to medical jargon, used excessively technical terms, or provided insufficient information. These findings are similar to those of a study conducted in Rio de Janeiro, which found that such an approach can generate insecurity, undermine trust in the care received, and interfere with patients’ autonomy and adherence to the therapeutic plan^(28)^.

It is important to highlight that, in cases of induced abortion, this issue is exacerbated by stigma, prejudice, discrimination, humiliation, and abuse by professionals, resulting in the absence or insufficiency of information and in care that does not meet quality standards^(26)^. In this care context, practices are not aligned with the National Humanization Policy, particularly regarding the principle of transversality and the guideline of expanded and shared clinical practice, which presuppose ongoing dialogue between users and health professionals, recognition of individual singularities, acknowledgment of users’ protagonism, and their participation in decisions related to care^(12)^.

As reported in other studies^(5,6,21,29)^, the accounts of most participants in this research reveal disrespect, abuse, and mistreatment that contradict the recommendations of the Ministry of Health outlined in the Technical Norm for Humanized Care in Abortion^(30)^, violate the NHP guideline on the care environment^(12)^, and constitute violence in abortion care, as they infringe upon women’s human rights^(14,31)^. Additionally, the results point to adverse outcomes resulting from the care provided, such as infection, the need for repeat curettage, and hysterectomy secondary to sepsis caused by retained placental tissue. These findings suggest clinical failures with significant physical and emotional repercussions, which may be considered near miss events^(32)^, given that maternal sepsis is an avoidable cause of mortality for thousands of women each year^(33)^, and the quality of the care environment is presumed to be a mediating factor in its prevention.

From another perspective, the women’s desire for private rooms, for care that protects their bodies, for prior explanations before examinations, and for opportunities to participate in decision making reveals that dignity, privacy, information, and autonomy in hospital care during the abortion process are fundamental to ensuring respectful, safe, and dignified care^(34)^.

Therefore, recognizing that abortion is a traumatic event-regardless of gestational age-and acknowledging the suffering of women who experience this process is part of ethical, sensitive, and technically safe care. When scientific recommendations are integrated with the principles of the NHP, such care contributes to reducing diagnostic and therapeutic delays, minimizing harm and the risk of complications, and promoting satisfaction with hospital care^(9,12,28,29,31,35)^. In this sense, the positive experiences reported by participants - marked by active listening, empathy, timely care, and psychological support, including support extended to partners - reinforce that it is indeed possible to humanize care even in adverse contexts.

However, this is not related to having or not having private health insurance, as among the ten women who had private coverage, only one reported having directly benefited from it by being able to choose where she would stay during hospitalization. This factor had a positive impact on her care experience during the abortion process, corroborating findings from Brazilian studies that highlight disparities in access to health services and qualified care^(36,37)^. Such inequities violate the SUS principle of equity and the recommendations of the National Humanization Policy, which advocate for welcoming and private environments for all, regardless of socioeconomic status. Moreover, disregarding the care environment as a component of humanized care exacerbates suffering, as documented in different regions of the country^(31)^ and in the present study.

As strategies to transform the characteristics of care in this context - which remains centered primarily on physical needs - there is a need to recognize abortion related complications, ensure appropriate clinical and obstetric management of these events, provide emotional support, and offer ethical guidance to women experiencing grief^(38)^. Furthermore, given the importance of implementing structured interventions for maternal and parental bereavement support^(21,39)^, initiatives for professional training and capacity building in this perspective should be encouraged, along with promoting women’s participation in decision making processes, as these aspects are associated with better care experiences in cases of spontaneous abortion^(21)^.

Contributing to the reconfiguration of care for women undergoing abortion, a study from Lithuania proposes that care should be structured around four pillars: qualified communication and information, with time for assimilating the situation; emotional support and assurance of privacy; a respectful and welcoming hospital environment that preserves privacy; opportunities for saying goodbye; and attention to emotional well being, including initial support, follow up assessments, and referral for psychological help when necessary^(40)^. These recommendations align with Brazil’s NHP and, at the same time, support the emotional processing of the experience and enable reflections on future life perspectives and reproductive intentions

To this end, it is essential to highlight the need for changes in health education, particularly in incorporating discussions on abortion and the abortion process, their ethical implications, conscientious objection, and the physical and emotional repercussions of this experience for women. Such discussions are fundamental for developing the technical and relational competencies^(35)^ required for care that is grounded in respect, empathy, and ethics-care that promotes safe, equitable, and humanized practices aligned with the principles and guidelines of the National Humanization Policy.

### Study limitations

Possible limitations of the study relate to the absence of software use in data analysis (researcher bias), the interval between the event and the interviews (recall bias), the different interview modalities (in person and remote), and the lack of articulation between participants’ perceptions and the diverse cultural, geographic, and economic contexts in which they received care during the abortion process. It is important to note, however, that these aspects did not compromise the depth of the analyses nor the interpretative consistency of the findings

### Contributions to the field of Nursing, Health or Public Policies

The study highlights the importance of active listening, sensitive welcoming practices, and clear, culturally appropriate communication between health professionals and women undergoing the abortion process as central elements of good obstetric practices, humanization, and dignified, respectful, and safe care. Furthermore, the lack of emotional support and the violations of the rights to dignity, information, privacy, and autonomy reveal shortcomings in hospital care, reinforcing the need for woman centered practices that are sensitive to the singularities of this moment.

## FINAL CONSIDERATIONS

The participants recalled hospital care during the abortion process with emphasis on negative experiences involving a lack of welcoming practices, ineffective communication, and impersonal interactions - elements that diverge from the principles of the National Humanization Policy. However, some women did feel welcomed and cared for, reflecting practices more closely aligned with humanized care guidelines.

Although there are established guidelines and recommendations for the humanization of obstetric care, the results indicate that these standards are only partially met, with persistent shortcomings that reveal violations of women’s human rights at a moment of profound vulnerability, particularly emotional vulnerability. By bringing these dimensions to light, the study contributes to strengthening evidence based best practices and improving the quality of care, offering insights that support ethical, respectful, and humanized care and, consequently, promote women’s comprehensive health - therefore reinforcing the implementation of Brazilian public policies.

## Data Availability

The research data are available within the article.
