# Supplementary material for: Women’s experiences regarding the care received in the hospital setting during the abortion process
Source: Rev Bras Enferm. 2026 Oct 16;79:e20250521. doi: 10.1590/0034-7167-2025-0521 (PMC13679158; doi:10.1590/0034-7167-2025-0521)
Supplement: Translation

# Translation: Experiências de mulheres sobre a assistência recebida no contexto hospitalar durante o processo de abortamento

**DOI:** 10.1590/0034-7167-2025-0521pt

**Authors:** Mariana Enumo Balestre, Iven Giovanna Trindade Lino, Maria Eduarda Pascoaloto da Silva, Juliana Amaral Prata, Telmara Menezes Couto, Viviane Cazetta de Lima Vieira, Erika dos Santos Ratuchnei Dal Pizzol, Sonia Silva Marcon

**Affiliations:** I Universidade Estadual de Maringá. Maringá, Paraná, Brasil; II Universidade Estadual do Rio de Janeiro. Rio de Janeiro, Rio de Janeiro, Brasil; III Universidade Federal da Bahia. Salvador, Bahia, Brasil

**Keywords:** Abortamento, Mulheres, Assistência Hospitalar, Humanização da Assistência, Enfermagem.

## Abstract

**Objetivos:**

apreender como mulheres rememoram a assistência recebida no contexto hospitalar durante o processo de abortamento.

**Métodos:**

estudo descritivo de abordagem qualitativa que utilizou como base conceitual a Política Nacional de Humanização. Os dados foram coletados entre maio e setembro de 2024, mediante entrevistas áudio gravadas, presenciais ou remotas, com 19 mulheres e submetidos à análise de conteúdo temática.

**Resultados:**

emergiram duas categorias: *Vivenciando uma assistência desrespeitosa*, que mostra a insatisfação com a forma de comunicação, informações insuficientes, desvalorização de queixas e do sofrimento emocional, ausência de privacidade e internação conjunta com gestantes; e *Experiências positivas no cuidado*, que incluem atendimento humanizado, apoio emocional e escuta ativa.

**Considerações Finais:**

as mulheres em geral rememoram a assistência recebida de forma negativa, o que aponta para a necessidade de adoção e implementação das boas práticas obstétricas e valorização das diretrizes da Política Nacional de Humanização na assistência durante o processo de abortamento.

## INTRODUÇÃO

O aborto é um evento relativamente comum na trajetória reprodutiva das mulheres^(1)^. No Brasil, configura-se como uma questão de saúde pública, haja vista o total de 2.258.104 internações por abortamento entre os anos de 2008 e 2018, representando uma média anual de 37,4 internações para cada 10 mil mulheres em idade fértil^(2,3)^. Não obstante, das 19.535 mortes maternas registradas no país no período de 2012 a 2022, 663 (3,39%) foram relacionadas ao aborto^(4)^. Esses dados evidenciam a magnitude do problema e impõem a necessidade de uma análise crítica acerca do modo como a assistência às mulheres em situação de abortamento tem sido conduzida.

Nesses casos, o atendimento hospitalar pode influenciar o processo de recuperação diante de atitudes e práticas que revelam negligências, discriminações e práticas desumanizadas, as quais, infelizmente, são comuns no contexto obstétrico^(2,5)^. A assistência às mulheres durante este processo deve contemplar dimensões clínicas, psicológicas, sociais e éticas^(6)^, uma vez que existem evidências de impactos significativos sobre a saúde física e mental^(7)^. A experiência do abortamento pode ser marcada por luto intenso e reações frequentes de luto severo - geralmente com diminuição ao longo do tempo^(8)^ - maior risco para ansiedade, depressão e transtorno de estresse pós-traumático, que são mais frequentes nos casos de abortos espontâneos recorrentes^(9)^.

Desse modo, considerando as repercussões do processo de abortamento sobre a saúde física e mental das mulheres, destaca-se a relevância de o atendimento hospitalar contemplar as dimensões clínicas, psicológicas, sociais e éticas. Revisão sistemática que incluiu estudos realizados na Ásia, África, Austrália e Europa constatou que muitas mulheres com histórico de aborto espontâneo desenvolvem transtornos mentais como ansiedade, depressão e estresse nas seis semanas seguintes ao evento e, ainda, que isto é mais frequentes em países de baixa e média renda^(10)^. Estes efeitos também foram observados em estudo realizado no Brasil, o qual identificou maior prevalência entre as primigestas e as mais jovens^(11)^.

Diante dessas questões, é preciso assegurar o direito das mulheres ao atendimento digno e ao cuidado ético, empático e resolutivo. Para tanto, o discurso normativo e programático do Ministério da Saúde (MS) brasileiro reforça a importância de considerar a pessoa em sua integralidade, reconhecendo suas necessidades emocionais, sociais e culturais como parte essencial de uma assistência à saúde integral. Para tanto, a Política Nacional de Humanização (PNH), de 2013, institui diretrizes que valorizam o acolhimento, a escuta qualificada, o vínculo entre profissionais e usuários, bem como a corresponsabilidade no cuidado^(12)^.

A despeito dessa política pública transversal do Sistema Único de Saúde (SUS), o atendimento humanizado em situações de abortamento ainda se configura como um desafio, seja por preconceitos dos profissionais, sobrecarga de trabalho, carência de capacitação adequada ou fragilidades na infraestrutura dos serviços^(13)^. Sendo assim, muitas vezes a assistência é limitada a uma abordagem clínica e procedimental, característica do modelo biomédico, desconsiderando as subjetividades e os aspectos sociais que permeiam a experiência do abortamento, com potencial para culminar em violências obstétricas^(13,14)^.

No Brasil, a produção científica na área se concentra, predominantemente, em análises epidemiológicas, normativas, e enfoque nas repercussões físicas e mentais do evento^(2,7)^. Embora fundamentais, esses estudos exploram de forma limitada como a assistência hospitalar durante o processo de abortamento é percebida pelas mulheres, o que limita a compreensão dos desencontros entre o atendimento preconizado nas políticas públicas e os cuidados efetivamente ofertados. Ao dar visibilidade às experiências vivenciadas por essas mulheres, o presente estudo contribui para o aprofundamento das dimensões subjetivas e relacionais do cuidado, subsidiando práticas assistenciais qualificadas, ações de educação permanente, e implementação das boas práticas obstétricas.

Este artigo é produto de uma dissertação de mestrado em Enfermagem defendida em março de 2025^(15)^.

## OBJETIVOS

Apreender como mulheres rememoram o atendimento recebido no contexto hospitalar durante o processo de abortamento.

## MÉTODOS

### Aspectos éticos

O estudo atendeu às prerrogativas das Resoluções nº 466/2012 e nº 510/2016 do Conselho Nacional de Saúde, bem como às orientações para pesquisas em ambiente virtual (Comissão Nacional de Ética em Pesquisa/2021), e foi aprovado pelo Comitê de Ética em Pesquisa com Seres Humanos da instituição signatária, (Parecer nº 6.656.413).

Todas as participantes registraram a anuência em participar da pesquisa no Termo de Consentimento Livre e Esclarecido (TCLE). No caso de entrevistas remotas, o TCLE foi previamente enviado por *WhatsApp* e devolvido em formato de arquivo digitalizado devidamente assinado. Para assegurar anonimato das participantes, na apresentação dos resultados do estudo, os extratos de suas falas estão identificados com a letra E, referente à entrevistada, seguida de um número indicativo da ordem de inclusão no estudo, e de outros relativos aos quantitativos de gestações (G), abortos (A) e filhos nascidos vivos (NV).

### Tipo de estudo e referencial teórico

Estudo descritivo, exploratório, de abordagem qualitativa, que utilizou como base conceitual a PNH. Essa abordagem permite explorar vivências e percepções subjetivas dos participantes, sobretudo em relação a temas sensíveis^(16)^, como é o caso do processo de abortamento.

A PNH, também conhecida como Humaniza SUS, propõe-se a subsidiar a concretização dos princípios do SUS no cotidiano dos serviços de saúde, para produzir mudanças nos modos de gerir e cuidar. No âmbito da assistência às mulheres durante a gestação, o parto e o puerpério, destacam-se algumas diretrizes fundamentais, a saber: acolhimento, ambiência, clínica ampliada e compartilhada, e defesa dos direitos dos usuários^(12)^. Cabe esclarecer que, para a elaboração do relatório de pesquisa, foram seguidas as recomendações do *Consolidated Criteria for Reporting Qualitative Research* (COREQ)^(17)^.

### Procedimentos metodológicos

As participantes do estudo foram selecionadas por conveniência, e a captação aconteceu por meio da consulta em prontuários, seguida da abordagem presencial na clínica ginecológica de uma instituição hospitalar pública com 30 leitos de obstetrícia e pronto atendimento para queixas obstétricas. Tendo em vista a baixa adesão (cinco mulheres), optou-se por estratégias adicionais, como divulgação da pesquisa em um grupo de apoio aos pais enlutados, com encontros quinzenais online coordenados por uma psicóloga (duas mulheres), e divulgação de um vídeo na rede social *Instagram* ^®^, com duração de um minuto, no qual a pesquisadora principal se apresentava e discorria sobre a motivação, os critérios e o modo de participação na pesquisa (12 mulheres). Em todos os casos, a pesquisadora compartilhou o seu número de telefone e endereço de e-mail, para contatos posteriores.

Os critérios de inclusão previamente definidos foram: ter 18 anos ou mais, ter recebido atendimento hospitalar durante o processo de abortamento, independentemente do tipo (provocado ou espontâneo), dos procedimentos realizados e do tempo transcorrido do evento. Por sua vez, não foram incluídas as mulheres que relataram não estarem emocionalmente preparadas para falar sobre o assunto.

No contato inicial, todas as possíveis participantes foram esclarecidas sobre os objetivos do estudo, motivação e tipo de participação desejada, sendo solicitado um número de telefone para o agendamento da entrevista àquelas que concordaram em participar. Das 26 mulheres contactas, cinco não foram incluídas por não se sentirem à vontade para rememorar a experiência, apesar do longo tempo transcorrido do evento (identificado em consulta ao prontuário), e duas não responderam às tentativas de contato posterior, totalizando 19 participantes.

### Coleta dos dados

Os dados foram coletados no período de maio a setembro de 2024, mediante entrevistas individuais semiestruturadas, agendadas em dia, horário e local de acordo com a preferência da participante e a disponibilidade da pesquisadora. Todas as entrevistas foram conduzidas por uma das autoras (enfermeira, solteira, mestranda em enfermagem, sem experiência na assistência às mulheres em processo de abortamento, mas com expertise em coleta de dados qualitativos), a qual não tinha nenhum tipo de relação/contato prévio com as participantes.

Cabe esclarecer que 11 entrevistas foram realizadas de forma presencial (nove no domicílio e duas em sala reservada na instituição hospitalar) e oito de forma remota por meio do *Google Meet* ^®^. Com a devida concordância, todas foram audiogravadas, utilizando recurso do celular (quando presencial) ou da plataforma de videoconferência (quando remota). Elas tiveram duração entre 30 e 105 minutos (média de 55 minutos), sem interrupções ou intercorrências que comprometessem a qualidade da gravação ou dos relatos. Cada participante foi entrevistada uma única vez, e aconteceram na presença apenas da participante e da pesquisadora, exceto com uma mulher, no domicílio, onde o companheiro estava presente, mas permaneceu a maior parte do tempo em outro cômodo da casa.

Para a realização das entrevistas, elaborou-se um roteiro, o qual foi testado com quatro mulheres, cujas entrevistas não foram incluídas no estudo, devido aos ajustes realizados no instrumento, que envolveram: i) acréscimo de perguntas sobre características sociodemográficas e de questões de apoio para valorização dos questionamentos do estudo e ii) reformulação de algumas questões que apresentavam enunciados em tom avaliativo. Após essas modificações, o roteiro ficou constituído por duas partes: a primeira com perguntas sobre as características sociodemográficas (idade, raça, estado civil, número de filhos vivos, número de abortos), e a segunda composta por uma questão norteadora: “Fale-me sobre como você vivenciou o processo de abortamento e como foi a assistência recebida nessa ocasião?”. Algumas questões de apoio também foram utilizadas quando necessário, a saber: “O que você gostaria que tivesse sido diferente durante este processo? Por quê? Especificamente em relação à assistência recebida, o que você aponta de positivo? E de negativo? Como foi a sua interação com a equipe médica e de enfermagem? E com as outras pacientes? Você participou de decisões relacionadas ao tratamento? De que forma? Você considera que teve liberdade para fazer perguntas e solicitar esclarecimentos? Tem mais alguma coisa que você gostaria de falar sobre sua experiência?”.

Ao término de cada entrevista, em local reservado, a pesquisadora gravou no celular suas impressões sobre o contexto e comportamentos não verbais (expressões faciais e corporais, entonação da voz, pausas, silêncios, choro). Preferencialmente no mesmo dia da realização da entrevista, o conteúdo e as observações da pesquisadora foram transcritos na íntegra com apoio do software *Transkriptor* ^®^. Durante este processo, não foram identificadas dúvidas que justificassem um novo contato com a participante. O material transcrito foi encaminhado apenas à participante que manifestou interesse em ler a transcrição para possíveis correções, exclusões ou acréscimos. A mesma, no entanto, não fez qualquer comentário ou sinalizou a necessidade de correções. Todas as demais declaram explicitamente não ser necessário ver o material transcrito, haja vista a utilização do recurso da gravação.

Para o encerramento da coleta de dados, adotou-se o critério de suficiência e exaustão dos dados^(18)^. A saturação teórica foi identificada na décima sexta entrevista, momento em que não emergiram novos códigos ou elementos analíticos relevantes para o fenômeno investigado. Para confirmar a repetição das informações e garantir a consistência dos achados, foram realizadas mais três entrevistas, totalizando 19 participantes, não havendo prejuízo à saturação teórica.

### Análise dos dados

As informações referentes à caracterização sociodemográfica e obstétrica foram organizadas em planilha no Excel e analisadas de forma descritiva, por meio de frequências absolutas. Para o tratamento dos dados qualitativos, não foi utilizado qualquer software, de modo que o conteúdo transcrito das entrevistas foi submetido à análise de conteúdo temática, conforme proposta de Bardin^(19)^, e desenvolvida em três etapas.

Na pré-análise, realizou-se a organização e familiarização com o material empírico por meio da leitura flutuante das entrevistas, com o objetivo de apreender o conjunto dos relatos e delimitar o corpus analítico. Nessa etapa, foram considerados os critérios de exaustividade, representatividade, homogeneidade e pertinência dos dados.

Na etapa de exploração do material, procedeu-se à identificação das unidades de registro presentes nas falas das participantes, que expressavam aspectos recorrentes das experiências relacionadas à assistência recebida durante o processo de abortamento. Esses trechos foram submetidos a um processo de codificação manual, sendo posteriormente agrupados em temas iniciais conforme a convergência de sentidos observada nos depoimentos. A partir desse processo analítico, os temas foram organizados em categorias analíticas, conforme apresentado no Quadro 1.

**Quadro 1:** Definição das unidades de registro, eixos temáticos e categorias analíticas do estudo

Unidades de Registro (n)	Temas	Categorias
- Primeiras impressões na chegada ao serviço (n=13) - Características do acolhimento inicial (n=14)	Acolhimento e abordagem inicial	Vivenciando uma assistência desrespeitosa e limitada durante o processo de abortamento
- Postura de normalização diante da ausência de batimentos cardio fetais (BCF) (n=3) - Manifestações de profissionais culpando a gestante (n=1) - Queixas sobre o atendimento médico (n=7)	Condutas profissionais impessoais e violências	
- Percepções sobre o compartilhamento do espaço físico com gestantes em trabalho de parto ou puérperas com seus bebes (n=7) - Descrições de episódios traumáticos explícitos(n=17)	Exclusão e sofrimento	
- Elogios sobre o atendimento recebido (n=6) - Menções à permanência em ala separada (n=1) - Vivências relacionadas ao cuidado multiprofissional (n=12)	Vivências acolhedoras e de cuidados sensíveis	Vivências acolhedoras e humanizadas em momentos de fragilidade

Por fim, na etapa de tratamento dos resultados, inferência e interpretação, realizou-se a análise crítica das categorias, buscando compreender os sentidos atribuídos pelas mulheres às suas experiências. Esse movimento analítico foi orientado pelos princípios e pelas diretrizes da PNH^(12)^, articulando os achados empíricos à literatura científica e ao contexto institucional da assistência hospitalar, permitindo identificar fragilidades e potencialidades no cuidado, bem como apontar caminhos para o aprimoramento da assistência durante o processo de abortamento.

Com o intuito de garantir a credibilidade, confiabilidade, confirmabilidade e transferebilidade, todo o processo de pesquisa, com ênfase na descrição metodológica foi registrado e está disponível para consultas futuras. Ademais, durante a análise, buscou-se identificar a coerência interna dos depoimentos, a recorrência temática e a consistência interpretativa.

## RESULTADOS

As 19 mulheres participantes do estudo possuíam entre 21 e 56 anos, das quais 17 referiram terem companheiro e 14 terem filhos vivos; 16 se declararam brancas e mais da metade (10) relatou atendimento por plano de saúde privado durante o processo de abortamento. Onze participantes residiam no município em estudo, cinco de outros municípios do estado do Paraná, duas em outros estados e uma em Osaka, no Japão.

O total de abortos referidos foi de 31 (28 espontâneos, um provocado e dois legais), sendo que o número de abortos por mulher variou de um a quatro. O tempo transcorrido desde o último aborto até o momento da entrevista variou de seis meses a 28 anos. Da análise das entrevistas emergiram duas categorias que serão descritas a seguir.

### Vivenciando uma assistência desrespeitosa e limitada durante o processo de abortamento.

Algumas mulheres relataram que o descontentamento com a assistência recebida teve início já na chegada ao serviço de saúde, no setor de acolhimento, quando é realizada a classificação de risco:

*Cheguei, a enfermeira da triagem perguntou quanto era meu sangramento e eu falei dois. Ela falou assim: “Ah, é pouco ainda! Então, vou te colocar como um verde”. E eu fiquei lá a manhã inteira esperando para ser atendida.* (E10 - G1/A1/NV0)

*Eu estava com sangramento ativo! Deixei isso bem claro. Pode ser que eu tenha visto coisa, mas parecia que a minha cor influenciou na forma que me trataram. Me colocaram como verde, com sangramento ativo e grávida de 5 semanas!* (E12- G1/A1/NV0)

Da investigação clínica à constatação do aborto, as falas revelam a preocupação das participantes em relação à comunicação com a equipe, que foi marcada pela impessoalidade da abordagem médica, pela falta de esclarecimentos ou pelo fornecimento de informações incompletas.

*Eu não senti que recebi informação suficiente e que me deram a segurança. “Olha, foi isso que aconteceu. A gente vai fazer a curetagem, não tem como não fazer ou coisa parecida”. Não me deram opção de escolha em momento algum.* (E15- G2/A2/NV0)

*Eu estava no Japão, só falaram que estava abortando. Fiquei na clínica até me darem alta.* […] *Não me falaram o que podia ter acontecido, não explicaram nada, só me deixaram lá até acharem que eu meus exames estavam bons para me darem alta.* (E16- G2/A1/NV1)

*Foi minha primeira gestação. Eu estava com meu esposo e o médico foi muito frio na maneira de falar. Na hora da ultrassom transvaginal, ele falou: “olha, sua gestação não evoluiu”. E mais nada. Nem consegui perguntar nada* [...] *A única coisa que me deram foi um papel sobre os riscos da cirurgia. Nem sabia que ia ser anestesia geral. Pensei que ficaria* acordada, mas apaguei*. Não faço ideia do que aconteceu naquela sala.* (E17- G1/A1/NV0)

A ausência de privacidade durante o atendimento, marcado pela presença de vários estudantes, de exames recorrentes, e ausência de orientações prévias à realização de procedimentos, fez com que as mulheres se sentissem incomodadas, violadas, expostas e desrespeitadas.

*O médico chegou e disse: “Abre as pernas!”. Eu fiquei, hã? Porque, de qualquer jeito, me senti violada! Até meu marido, que sentado na poltrona, se assustou.* (E6- G4/A2/NV2)

*Eu fiquei, acho que uns 40 minutos, na posição ginecológica. Um vinha e olhava* […]*. Até por ser um hospital universitário! Vinha outro e mais outro* […]. *Era desconfortante!* (E5- G5/A3/NV2)

*Eu presenciei algumas coisas que são chatas. Não só pelo fato de ser mulher e gestante. Acho que por estar no hospital, deveria ser tratada com respeito, além de privacidade, que eu não tive. Fui atendida com várias pessoas na sala.* (E14- G2/A1/NV1)

O fato de permanecerem internada aguardando a realização de procedimentos, como a curetagem ou a aspiração manual intrauterina (AMIU), na mesma enfermaria com gestantes em trabalho de parto ou puérperas com recém-nascidos vivos, constitui fator gerador de sofrimento para as participantes.

*Aquela noite foi angustiante para mim porque naquela sala só tinha mulheres grávidas. Então, tinha mulheres que estavam escutando o coração do neném, outra que estava com aquele aparelhinho que já ia para a cesárea* […]. *Aquilo acabou comigo! Eu só chorava porque eu ia fazer uma cirurgia para tirar e elas iam ter o neném.* (E17- G1/A1/NV0)

*Eu olhava para o lado, tinha uma grávida, olhava para o outro, tinha uma mulher com um barrigão, ou com uma criança, e eu sozinha* […]. (E12- G1/A1/NV0)

[...] *eu estava na ala da maternidade e toda hora passava uma pessoa perguntando quando que eu ia ganhar. Eu já sabia que eu não tinha mais nada dentro de mim*. (E18- G5/A4/NV1)

Ademais, nas situações de aborto ilegal, o constrangimento e o medo foram muito presentes desde a chegada no hospital.

*Foi uma decisão bem difícil. Não é algo aceito, é ilegal e tudo mais. Não sabia se teria algum problema, mas, para mim, realmente não fazia sentido naquele momento. Então, além da escolha, tinha a preocupação de estar na frente de um médico, tendo que mentir, lidando com toda aquela situação* […]. *Querendo ou não, a gente nunca sabe quem vai encontrar* […]. (E11- G1/A1/NV0).

Além dos sentimentos negativos experienciados, algumas entrevistadas relataram negligências no atendimento que culminaram em desfechos desfavoráveis, gerando mais sofrimento.

*Não fizeram a curetagem correta porque disseram que o meu útero estava bastante inchado. Fiquei internada porque eles queriam fazer curetagem novamente. Porém, depois desse atendimento, eles não fizeram outro ultrassom para saber como é que estava. Se realmente tinha limpado, se a placenta toda tinha saído […]. Eu acabei perdendo meu útero porque a placenta infeccionou dentro de mim. Tive hemorragia, infecção generalizada e acabei perdendo meu útero.* (E2- G1/A1/NV0)

*Eu estava com muita infecção e com o resto da placenta. Não tinha expelido tudo porque o meu colo de útero tinha fechado de novo. Tive alta, mas precisei voltar no hospital, para fazer mais exames e mais uma curetagem. Todo aquele processo agonizante de novo!* (E1- G2/A1/NV1)

*Antes do terceiro aborto, não foi feito ultrassom porque o médico que fazia não estava no hospital e ele achou que não havia necessidade. Eu perdi esse, de novo* […]. (E4- G4/A4/NV0)

### Vivências acolhedoras e humanizadas em momentos de fragilidade

Algumas mulheres rememoraram ter recebido uma assistência humanizada, expressa no acolhimento, no cuidado sensível e nas orientações que receberam acerca do processo de abortamento:

*Ela fez o exame de toque e falou: “O colo do útero está aberto!”. Eu pensei “não é menstruação!” e falei: “Está acontecendo alguma coisa, doutora? Tem alguma coisa errada?”. Ela falou para eu me vestir primeiro. Hoje, vejo isso como um sinônimo de cuidado, certo?* (E18- G5/A4/NV1)

*Mais uma vez, eu estava lá no hospital. Fui acolhida de um jeito que nunca tinha sido. Era a 4ª gestação, o pessoal já me conhecia, todas as enfermeiras falavam comigo* […]. *Foi tudo muito gostoso! Eu fiquei lá um pouquinho com elas e foi muito gratificante para mim. Além disso, eu tive muito acolhimento do médico que fez o ultrassom. Ele foi muito didático.* (E4- G4/A4/NV0)

As percepções positivas estiveram relacionadas com as manifestações de disponibilidade e empatia, escuta ativa e atitudes de respeito por parte dos profissionais, além de fornecimento de informações, esclarecimento de dúvidas e atendimento psicológico durante a internação:

*Toda hora vinham ver se eu estava bem. Teve uma médica que deu super apoio para mim na hora que eu precisei. A enfermeira também. Elas conversaram bastante comigo. Inclusive, a médica falou que ia ver meus exames mais rápido para eu não ter que ficar aqui muito tempo. Ia agilizar minha alta* […]. *Senti que me deram todo o apoio que precisava.* (E8- G1/A1/NV0)

*As enfermeiras foram bem sensíveis. Quando o anestesista chegou eu falei: só não quero que amarre meus braços, quero colocar as mãos na cabeça. Aquela posição me lembrava a curetagem anterior. E ele deixou, foi muito compreensivo, não vou me esquecer disso jamais*. (E9- G2/A2/NV0)

*A psicóloga ia conversar comigo todos os dias!* […] *ela vinha com palavras, mas também para me ouvir. Ela ficou bastante tempo com meu marido cuidando dele*. (E2- G1/A1/NV0).

O local de internação, seja em setores externos à maternidade ou em quartos separados do contato com outras gestantes e puérperas, configurou-se como um determinante para uma vivência menos traumática.

*Eu tinha que esperar até o outro dia para fazer o ultrassom. Não me deixaram na maternidade porque eles achavam que eu ia perder*. (E2- G1/A1/NV0)

*Eu fiquei na ala de maternidade, mas, ao meu lado, estava uma mulher que tinha retirado o útero Graças a Deus não era gestante e nem puérpera*. (E4- G4/A4/NV0)

*Eu disse para a médica “Não quero ficar na ala da maternidade” e ela falou “Não tem jeito porque para fazer a indução do seu parto, você tem que ficar”. Tinha aquele monte de grávida e eu comecei a falar: “Me tira daqui!”. Como tinha plano privado, pude ir para outro hospital, onde não fiquei na maternidade. Foi a melhor decisão que eu poderia tomar no momento*. (E1- G2/A1/NV1)

## DISCUSSÃO

As mulheres deste estudo, de modo geral, rememoraram que a assistência hospitalar recebida durante o processo de abortamento se caracterizou por demora no atendimento inicial, impessoalidade na abordagem profissional, problemas na comunicação, exposição dos seus corpos, ausência de esclarecimentos e de consentimento prévio à realização de exames e procedimentos, negligência, e sofrimento decorrente da permanência em enfermarias compartilhadas com gestantes ou puérperas. Ou seja, para as mulheres, o processo de abortamento foi marcado não apenas pela perda gestacional, mas também por experiências negativas no atendimento, que ampliaram a dor e o sofrimento vividos.

Tais achados vão de encontro às diretrizes de acolhimento, escuta qualificada, garantia de privacidade e corresponsabilidade no cuidado preconizadas na PNH^(12)^. Ao mesmo tempo, revelam situações de desrespeito aos direitos das mulheres à dignidade, à informação, à privacidade e à autonomia, assim como apontam para a invisibilidade dos aspectos subjetivos que perpassam essa vivência da vida reprodutiva feminina, sobretudo considerando que a maioria das participantes teve mais de um aborto espontâneo.

Nestas ocasiões, sabe-se que o apoio emocional e as relações estabelecidas com os profissionais de saúde podem influenciar positiva ou negativamente a experiência do abortamento^(20)^. Corroborando com os resultados desta pesquisa, uma revisão sistemática constatou que a insatisfação das mulheres com o atendimento prestado em serviços de emergência da Pensilvânia e de diferentes regiões dos Estados Unidos deve-se, em parte, à falta de apoio emocional^(21)^.

Tendo em vista que o suporte recebido durante o processo de abortamento molda a experiência vivida e o impacto psicológico associado^(9)^, é importante que os profissionais ponderem o desconhecimento das mulheres sobre os parâmetros utilizados para classificação de risco (volume de sangue perdido, frequência cardíaca, pressão arterial, idade gestacional e intensidade da dor), informando-as que, apesar de as gestantes com sangramento terem prioridade para o atendimento^(22)^, o tempo de espera não agravará seu quadro clínico. Porém, considerando que o acolhimento envolve reconhecer as necessidades singulares e o contexto de vida dos usuários, ao negligenciar essa diretriz, o serviço reforça a aplicação mecânica de normas técnicas e o desenvolvimento de práticas reducionistas que potencializam as repercussões psíquicas da perda gestacional^(12)^.

Nesse sentido, um estudo de revisão sistemática identificou que mulheres da Ásia, da Europa e da África referiram altos níveis de ansiedade, depressão e estresse relacionado à vivência do aborto espontâneo^(10)^. Entre as norueguesas, observou-se maior sofrimento psíquico após o aborto espontâneo em curto e médio prazos, enquanto o aborto induzido esteve associado, a longo prazo, a escores mais elevados de evitação, culpa, vergonha e alívio^(23)^.

Por essas questões, a comunicação clara entre profissionais e pacientes se mostra fundamental pois favorece a coleta de informações relevantes para a construção do plano terapêutico, proporciona bem-estar e fortalece os princípios éticos da não maleficência e da autonomia, desde que seja sustentada por escuta ativa, linguagem não estigmatizante e reconhecimento do sofrimento e dos direitos das mulheres^(24-27)^. Essas características não foram identificadas pelas participantes deste estudo na interação com os profissionais, visto que, por vezes, estes recorreram a jargões médicos, utilizaram termos excessivamente técnicos ou forneceram informações insuficientes. Tais achados são semelhantes aos encontrados em uma pesquisa realizada no Rio de Janeiro, a qual verificou que essa abordagem pode gerar insegurança, comprometer a confiança na assistência recebida e interferir na autonomia das pacientes e na adesão ao plano terapêutico^(28)^.

É importante destacar que, nos casos de aborto induzido, essa problemática é potencializada por estigmas, preconceitos, discriminações, humilhações e abusos por parte dos profissionais, culminando na ausência ou na oferta insuficiente de informações, bem como em cuidados que não atendem aos padrões de qualidade^(26)^. Nesse contexto assistencial, as práticas não estão em consonância com a PNH, especialmente no que tange ao princípio da transversalidade e à diretriz da clínica ampliada e compartilhada, que pressupõem o diálogo constante entre usuários e profissionais de saúde, a valorização das singularidades da pessoa, o reconhecimento do seu protagonismo e a sua participação nas decisões relativas ao cuidado^(12)^.

Assim como relatado em outras pesquisas^(5,6,21,29)^, as falas da maioria das participantes deste estudo evidenciam desrespeitos, abusos e maus-tratos, que contrariam as recomendações do MS dispostas na Norma Técnica de Atenção Humanizada ao Abortamento^(30)^, infringem a diretriz de ambiência da PNH^(12)^ e configuram violências na assistência ao processo de abortamento, pois violam os direitos humanos das mulheres^(14,31)^. Ademais, os resultados também apontam para desfechos adversos advindos da assistência, como a infecção, necessidade de nova curetagem e histerectomia secundária à sepse por restos placentários, sugerindo falhas clínicas, com importantes repercussões físicas e emocionais, as quais podem ser consideradas como *near miss* ^(32)^, visto que a sepse materna é uma causa evitável de mortalidade em milhares de mulheres por ano^(33)^, e presume-se que a qualidade do ambiente de cuidado constitui elemento mediador nessa prevenção.

Em outra perspectiva, o desejo das mulheres desta pesquisa por quartos privativos, por atendimentos que resguardem seus corpos, por esclarecimentos prévios à realização de exames e por oportunidades de participação nas decisões desvela que a dignidade, a privacidade, a informação e a autonomia na assistência hospitalar durante o processo de abortamento são fundamentais para a efetivação de cuidados dignos, respeitosos e seguros^(34)^.

Desse modo, reconhecer que o abortamento é um evento traumático, independentemente da idade gestacional, e acolher o sofrimento das mulheres que vivenciam esse processo faz parte da assistência ética, sensível e tecnicamente segura que, ao incorporar as recomendações científicas aos princípios da PNH, contribui para reduzir atrasos diagnósticos e terapêuticos, minimizar danos e riscos de complicações, bem como para promover a satisfação com a atenção hospitalar^(9,12,28,29,31,35)^. Nesse sentido, as experiências positivas narradas pelas participantes deste estudo, as quais perpassaram por escuta ativa, empatia, agilidade no atendimento e apoio psicológico, inclusive com extensão ao companheiro, reforçam que é possível humanizar o cuidado mesmo em contextos adversos.

No entanto, isto não guarda relação com o fato de possuir ou não plano de saúde, pois das dez mulheres que tinham plano privado, apenas uma relatou ter se beneficiado diretamente desse recurso, ao poder escolher o local de permanencia durante a internação. Esse fato repercutiu positivamente em sua experiência assistencial durante o processo de abortamento, corroborando dados de estudos brasileiros que mostram as disparidades de acesso aos serviços de saúde e à assistência qualificada^(36,37)^. Essa condição fere o princípio da equidade do SUS e as recomendações da PNH, que defendem ambientes acolhedores e privativos para todos, independente de condição socioeconômica. Além do mais, ignorar a ambiência como um componente do cuidado humanizado agrava o sofrimento, como já registrado em diferentes regiões do país^(31)^ e no presente estudo.

Como estratégias para modificar as características da assistência nesse âmbito, a qual permanece centrada nas necessidades físicas, têm-se o reconhecimento das complicações relacionadas ao abortamento, o manejo clínico e obstétrico dessas intercorrências, a disponibilização de apoio emocional e o oferecimento de orientação ética às mulheres que vivenciam o luto^(38)^. Ainda, compreendendo a relevância da implementação de intervenções estruturadas de apoio ao luto materno e parental^(21,39)^, deve-se fomentar iniciativas de formação e capacitação profissional nessa perspectiva, aliadas ao incentivo da participação da mulher nos processos decisórios, visto que essas questões se relacionam com melhores experiências no atendimento recebido em casos de aborto espontâneo^(21)^.

Corroborando para a reconfiguração da assistência às mulheres em processo de abortamento, uma pesquisa da Lituânia propõe que os cuidados sejam estruturados em quatro eixos, a saber: comunicação e informação qualificadas, com tempo para assimilação da situação, apoio emocional e garantia de privacidade; ambiente hospitalar respeitoso, acolhedor e com preservação da privacidade; oportunidade para a despedida; e atenção ao bem-estar emocional, com apoio inicial, avaliação nos retornos e encaminhamento para ajuda psicológica quando necessário^(40)^. Tais recomendações se mostram alinhadas à PNH do Brasil e, ao mesmo tempo, favorecem a elaboração emocional da experiência e possibilitam reflexões sobre perspectivas futuras sobre a vida e as intenções reprodutivas.

Para tanto, destaca-se a necessidade de mudanças na formação em saúde, no sentido de incorporar debates acerca do aborto e do processo de abortamento, de suas implicações éticas, da objeção de consciência, das repercussões físicas e emocionais dessa experiência para as mulheres, com vistas ao desenvolvimento de competências técnicas e relacionais^(35)^ condizentes com uma assistência baseada no respeito, na empatia e na ética, que impulsiona cuidados seguros, equitativos e humanizados, alinhados aos princípios e às diretrizes da PNH.

### Limitações do estudo

Possíveis limitações do estudo relacionam-se à não utilização de software na análise dos dados (viés do pesquisador), ao intervalo entre o evento e a realização das entrevistas (viés de esquecimento), às diferentes modalidades de entrevista (presencial e remota), e à ausência de articulações entre as percepções e os distintos contextos culturais, geográficos e econômicos nos quais as mulheres foram assistidas durante o processo de abortamento. Ressalta-se, contudo, que tais aspectos não comprometeram a profundidade das análises, nem a consistência interpretativa dos achados.

### Contribuições para a área da Enfermagem, Saúde ou Política Pública

A pesquisa destaca a importância da escuta ativa, do acolhimento sensível e da comunicação clara e culturalmente apropriada entre os profissionais de saúde e as mulheres em processo de abortamento enquanto elementos centrais das boas práticas obstétricas, da humanização e do cuidado digno, respeitoso e seguro. Além disso, a falta de apoio emocional e os desrespeitos aos direitos à dignidade, à informação, à privacidade e à autonomia evidenciam falhas na atenção hospitalar, reforçando a necessidade de práticas assistenciais centradas na mulher e sensíveis às singularidades desse momento.

## CONSIDERAÇÕES FINAIS

As participantes rememoram a assistência hospitalar durante o processo de abortamento com ênfase, em experiências negativas que envolveram falta de acolhimento, comunicação ineficaz e impessoalidade na assistência ofertada, elementos que se distanciam dos princípios da PNH. No entanto, algumas se sentiram acolhidas e cuidadas, aproximando-se das diretrizes do cuidado humanizado.

Embora existam diretrizes e recomendações para a humanização da assistência obstétrica, os resultados apontam que essas normativas são parcialmente atendidas, persistindo falhas que denunciam desrespeitos aos direitos humanos das mulheres em um momento de grande vulnerabilidade, sobretudo emocional. Ao evidenciar essas dimensões, o estudo contribui para o fortalecimento das boas práticas baseadas em evidências e para a qualificação da assistência, ao oferecer subsídios para a promoção de cuidados éticos, respeitosos e humanizados e, por conseguinte, para a saúde integral das mulheres, corroborando assim para a efetivação das políticas públicas brasileiras.

## Data Availability

Os dados de pesquisa estão disponíveis no corpo do artigo.
